# The Week's Work

**Published:** 1911-03-25

**Authors:** 


### The Week's Work.

### THE LONDON HOSPITAL.

In our issue of March 11 last appeared a letter from Mr. Sydney Holland referring to a reply made by us to a correspondent who had desired to know whether or not the London Hospital was the largest hospital in the world. We ventured to state that the London Hospital is the largest voluntary hospital in the British Empire, but Mr. Holland submitted that this statement is not accurate, since the Edinburgh Infirmary possesses more beds than does the East End institution, and he added that he has " always been careful to claim for the London that it is merely the largest (voluntary) hospital in England." We intimated in a footnote to the letter that the opinion of the Edinburgh authorities would be interesting with regard to the subject; since then we have received through the courtesy of Dr. Warburton detailed statistics regarding the Edinburgh Infirmary. It is interesting to compare these with the similar statistics furnished by the London:—

| | Number of beds (1911) | Number of In-patients (1910) | Out-patients (1910) | Daily average of occupied beds (1910) | Average stay per night |
|---|---|---|---|---|---|
| Edinburgh Royal Infirmary | 942 | 12,342 | 38,9[illegible]0 | 842 | 25·1 days |
| London Hospital ... | 922 | 15,2[illegible]7 | 2[illegible]2,822 | 803 | 20·21 ,, |

These figures speak for themselves, and they show that while we were undoubtedly wrong in stating that the London Hospital is the largest voluntary hospital in the Empire so far as number of beds is concerned, we are correct in designating it as such when the fact is taken into account that it treats annually a far larger number of patients than does any other voluntary hospital in the Empire—the large Indian institutions not excepted. The tremendous superiority in numbers of the London out-patients and the larger number of its in-patients give it a right to claim the distinction that it is the largest hospital, for we do not think that the majority of our readers will join issue with us in disregarding as a final criterion, the number of beds by itself. As a matter of fact, other points have to be taken into consideration in speaking in superlatives: the extent of ground covered by hospital buildings, the number of staff, and the cost of administration. An interesting fact shown by the above statistics is the increased average time patients were under treatment at the Infirmary, as compared with the corresponding average at the London. In Scottish hospitals this average is, on the whole, slightly higher than in English hospitals—a fact which is worth some attention and to which we hope to refer at length in another issue—but the difference between these two hospitals is very striking when their accommodation for in-patients is taken into account. It seems to show that the work done at the London is greater than at the Edinburgh—a reflection which is strengthened by the figures in the second column. We merely based our original statement on the work accomplished, and, looking at that point only, it seems to us that we were right in saying that the London is the largest voluntary hospital in the Empire.

### "HOSPITAL NIGHT" AT THE THEATRES.

The unfortunate difference of opinion over the money derived from cinematograph shows on Sunday, which was revealed in our report on the meeting of the Sunday Fund last week and on which we comment in a leading article in this number, has led to the repetition of an old suggestion. This we give in the words of its author, Sir T. W. Snagge, who sent it to the *Times*: " Why should we not have, say, ' Hospital night ' in our theatres, in which a collection between the acts, made simultaneously at all places of amusement throughout the country, might at least be tried as an experiment?" The theatrical profession has done so much for hospitals that there is little doubt that its assistance would be granted. Nor is it likely that the collecting-box would make the theatres unpopular on one night in the year. Such suggestions, however, are more complicated than they appear to be at first sight. It will be amusing if its adoption is the only result of the partizan outcry against Sunday cinematograph shows.

### UNIVERSITY COLLEGE HOSPITAL APPEAL.

It is most satisfactory to learn that the special appeal for £10,000, issued exactly a year ago by University College Hospital, has been successful. The debt has been paid off, and the new year started with a balance in hand. Still, the public must be impressed with the fact that the same state of things can only recur if a large number of annual subscribers be not forthcoming. The unfortunate tendency of people to refuse annual subscriptions to anything except their clubs has led to the adoption of the appeal system, which, besides being a great strain upon the hospitals' institutional staffs, presents greater difficulties every time it is resorted to, owing to the indifference of people accustomed, in fact inured, to constant demands for large sums of money. Now University College Hospital has found its financial feet again, we hope its income will continue to be raised steadily. Last year was a record year for successful appeals, and this great hospital certainly deserved its share in the results.

### THE ROYAL INFIRMARY, EDINBURGH.

We have received from the author, Fleet-Surgeon W. E. Home, R.N., an interesting pamphlet on the Administration of the Royal Infirmary, Edinburgh. The matter does, it is true, consist only of modest notes, yet these are set out in a readable fashion and form a record of the successful organisation of this large institution as it appeared in 1908. In the future these notes will be of the greatest value and interest for comparison, while for the present the pamphlet should be a convenient source of information and hints to hospital administration. For the small sum of sixpence a very good idea can be obtained from these notes of the working of the wheels of a well-ordered and economically run hospital. The results of trials of various materials for floors, etc., are briefly mentioned and in many other details we are given the benefit of practical experience. The notes on the victualling of this populous institution take up an important proportion of the pamphlet; the lines on which this department is run are followed more or less closely by many other institutions in the country generally. The author is, of course, not pretending how things should be done, but simply recording actual existing methods; and therein lies the usefulness of this record.

### A FRENCH INSTITUTE FOR PUERICULTURE.

Professor Pinard, at a recent meeting of the Paris Academy of Medicine, raised the question of the establishment of Institutes of puericulture. As an example of the kind of institute which he thought ought to be founded in all towns where there existed a Normal School for female teachers (Ecole Normale d'Institutrices), he cited that of Porchefontaine, near Versailles, founded by the Society "La Pouponnière," directed by Madame Veil-Picard. In that establishment each girl-mother is received and supported for two years on condition of suckling one of the infant pensioners of the Society in addition to her own child. With this principal home there is connected a working colony, called "Les Nids de Porchefontaine," in which the working women of Versailles and district bring up other children by hand. The necessary milk is obtained from a model cow-house, which is rigorously inspected in order that, as far as possible, the milk may be consumed without danger in a raw condition. A school of domestic economy, a temporary infirmary, and daily consultations for nurslings complete this remarkable foundation. The results obtained so far have been entirely satisfactory. In ten years the number of deaths out of 1,946 children admitted has only amounted to 54. Students of the normal schools for female teachers at Versailles and Sèvres are allowed to visit the establishment and so obtain the knowledge necessary for them in the teaching thereafter of the essentials of puericulture. The Professor's views were unanimously adopted by the members of the Academy.

### A WANT IN HOSPITAL LITERATURE.

Hospital literature is, like that of other departments of human activity, increasing at a tremendous rate. In the vast mass of things published that has accumulated during the past decade, books and reports dealing with hospitals figure largely, and yet there are one or two subjects connected with hospitals which stand in need of further attention. We have lately had several inquiries, for instance, about books dealing with hospital administration abroad, as distinct from administration in this country. At the present moment no book on the subject exists in the English language which gives an adequate summary of hospital methods on the Continent. The student who wishes to investigate the subject must have recourse to scattered articles and résumés given in various volumes, unless he is able to read French and German and Italian—with, as far as the Russian hospitals are concerned, that exceedingly extravagant language spoken by the Slavs—in which case he will find several exhaustive works on the subject. There seems thus an opening for a text-book on hospital administration that shall collate the various scattered contributions in the literature and collect the best of them into a detailed essay on the subject. We commend the want to the attention of those of our readers who have the energy and the interest to undertake the compilation of such a work. It is bound to find a reading public, and its usefulness is likely to be extreme.

### A COMPARISON OF DIFFERENT METHODS.

It is only by the comparison of separate methods of administration that we can select the best, and a review of our own schemes alongside those adopted by co-workers in other parts of the world is bound to be of great practical value. What is wanted in such a summary is a clear and accurate digest of method, with the why and the wherefore definitely stated. To take only one point. We are in the habit of imagining that continental hospitals are seriously understaffed, so far at least as the nursing personnel is concerned. Continental administrators, on the other hand, maintain that our nursing staffs are extravagant. It would be worth while to attempt a full inquiry into the subject, and to arrive at some definite conclusion with regard to the matter. Nothing is more striking to the English visitor accustomed to a large nursing staff, than to find the amount of work achieved by a comparatively small staff at a big Russian or German clinic. To be able to judge the matter fairly, it is necessary to take into consideration the different methods of administration, and to compare them on their merits. That has not as yet been done for the methods that have been adopted during the last ten years. Sir Henry Burdett's monumental work affords a reliable basis for comparison, but since it was published many new methods of administration have been accepted on the Continent and in America. For example, the question of hospital abuse has exercised the minds of hospital workers very seriously during the past decade, and, as we have already shown in special articles dealing with individual hospitals in Germany and Holland, some particular methods have been adopted, while at the present moment even more drastic methods are under consideration at some institutions.

### THIS WEEK'S DRUG MARKET.

Business has been unusually quiet during the week, but there seems no reason to expect that the quiet period which has set in will be unduly prolonged. Changes in value are few in number. Cod-liver oil continues to be quoted at high rates which have a tendency to advance rather than to recede, in consequence of further unfavourable reports from the Norwegian fishing grounds. Menthol is in slightly better demand and the price has ceased to decline, at any rate, for the present. A fair business has been done in oil of aniseed at good prices. Cream of tartar is a shade cheaper, owing to the absence of demand. Jalap is tending dearer. The price of opium is, if anything, rather lower, as also is the price of morphine.

